# Critical conversations: a user-centric approach to chatbots for history taking in the pediatric intensive care unit

**DOI:** 10.3389/fped.2025.1646989

**Published:** 2025-08-12

**Authors:** Candace Collins, James Fackler, Melissa Jerdonek Sacco, Maia Jacobs

**Affiliations:** ^1^ Department of Anesthesiology and Critical Care Medicine, Johns Hopkins Hospital, Baltimore, MD, United States; ^2^ Department of Pediatrics, University of Virginia Health Children's, Charlottesville, VA, United States; ^3^ Department of Computer Science, McCormick School of Engineering, Northwester University, Evanston, IL, United States; ^4^ Department of Preventive Medicine, Feinberg School of Medicine, Northwestern University, Chicago, IL, United States

**Keywords:** PICU (pediatric intensive care unit), chatbot adoption, communication, clinical reasoning, diagnostic reasoning

## Abstract

In this article, we describe the potential utility and design of chatbots to improve history taking in the pediatric intensive care unit (PICU). The fast-paced, high-stakes environment of the PICU often forces clinicians to obtain only enough information to make immediate clinical decisions. Specific barriers to comprehensive history taking include insufficient time, frequent interruptions, caring for a wide range of conditions, need for timely interventions, and language differences. We propose that chatbots could play a critical role in improving history taking in the PICU by collecting information related to a patient's current presentation and exploring areas that are commonly neglected, such as social histories. To explore the use of chatbots in the PICU setting, we will first describe the current scope of chatbots as medical history taking aids. Next, we will outline specific considerations for the development of chatbots for the PICU, including methods for involving users, such as patients, caregivers, and clinicians directly in the design, mitigating false information, and establishing safeguards for chatbot behavior. Finally, we will review methods to evaluate chatbots. The overall purpose of this perspective article is to 1) propose the PICU as a novel environment where chatbots could improve history taking and diagnostic reasoning and 2) delineate specific user-centric design and evaluation methods.

## Introduction

Every medical student learns that history-taking is the key to diagnosis (1). Diagnostic decision making relies on two types of Thinking: Type 1, which is intuitive pattern-recognition, and type 2, which is a slower, more analytical approach to solve complex problems (2). Insufficient time favors type 1 thinking in acute care settings where urgency demands fast thinking and timely intervention (3–5). PICU clinicians primarily rely on hypothesis-driven reasoning to gather information, spending more time testing these hypotheses than conducting more open-ended, patient or caregiver-paced discussions. Insufficient time, a wide range of conditions, fatigue, and language barriers can interfere with physicians accessing Type 2 processing (6).

Another consequence of Type 1 dominant history-taking is the lack of attention to social determinants of health (SDHs) and social needs. This bias affects outcomes for critically ill children (7–9). Adding further complexity, patients in the PICU range from infants to young adults with varying disease processes, often within a single patient. The broad range of patient presentations and complex medical environment with frequent and necessary interruptions increase the risk for diagnostic error through the inability to obtain complete information (4, 6).

We propose history-taking chatbots for the PICU to augment gathering and collating comprehensive histories, particularly areas commonly neglected, like social history. The chatbot could gather information at any point during a hospital stay, allowing families to share more information while also taking the time to explore information related to SDHs and social needs. Chatbots with generative AI capacity could use this information to enhance clinicians' decision-making. In this article, we will outline the current uses of chatbots as medical history-taking aids. We then propose a potential user-focused research agenda and design considerations for chatbots in the PICU.

## Chatbots as medical history-taking tools

Chatbots were introduced for clinical use over four decades ago, but their adoption has accelerated with the accessibility of publicly available models like ChatGPT (10–12). These tools can analyze large amounts of data in seconds, supporting time-sensitive decisions. To date, most studies evaluating chatbots for medical history taking are focused on the outpatient setting for specific areas of the history, allergies, symptom monitoring, or cancer risk (12–18). One small feasibility study evaluated the use of an AI chatbot for comprehensive medical history taking, including SDHs (19). In the PICU, decision-making requires multiple components of the history and further studies are needed to evaluate a chatbot's utility in this environment.

The use of large language models (LLM) in intensive care unit (ICU) settings at this time focus on computing large amounts of data to aid in risk stratification, early warning systems, and patient education (20–22). In pediatrics, chatbots have been developed for increasing access to mental health support, patient education, and improving appointment adherence (23–27). Growing familiarity with generative AI tools, like ChatGPT, is increasing user comfort with using these tools for health-related issues (28). Chatbots have high potential to be useful in ICUs as history-taking tools and diagnostic aids, but this has largely been unexplored. In this article, we discuss the use case of chatbots as comprehensive history taking tools that interact directly with caregivers/patients and improve diagnostic reasoning. The focus of this article is on user-centric design and research considerations, particularly for patient/caregiver and medical team engagement.

## Patient/caregiver and medical team involvement in design

Over the past several decades, there has been a paradigm shift from medical paternalism to patient autonomy and shared-decision making between patients and physicians (29). As clinicians now support patient involvement in healthcare decisions, AI developers must empower patients to be active participants in the development of healthcare AI instruments. Best practices about how to involve users (i.e., patients/caregivers and clinicians) in the design of clinical AI tools are lacking. User-focused AI designed methods are referred to as “participatory AI design.” This is a critical approach to ensure that these tools represent the needs of a population while supporting equity and inclusivity, particularly for marginalized populations (30). A 2023 article by Delgado et al. describes the current state of participatory AI design as more “consultative” where there are specific preferences or values that developers try to elicit from participants. They delineate scaled “modes of participation” for AI design, which include *consult*, *include*, *collaborate*, and *own*. It is important to determine the “dimension of participation,” which can be elicited by four questions (why is the participation needed; what is on the table; who is involved; and what from does stake holder participation take) to determine the goals, scope, and methods of the project for participants (31).

Ideally, patients/caregivers and members of the medical team (i.e., physicians, nurses, and social workers) will start at the “collaborate” mode of participation for chatbot design. Patient/caregivers and clinicians will co-create and co-evaluate the tools with developers, expanding beyond simply eliciting specific preferences (31). Recruitment should target patients and families of diverse backgrounds who have previous experience with the PICU and are not current patients due to the high-stress nature of these admissions. Social media could be used as a potential recruitment tool in addition to providing information to pediatricians to distribute to the community (32). The design sessions should offer a mix of on-line and in-person collaboration. It would also be most beneficial to have a “core” group of participants present throughout the design process (32). To engage children, developers could consider novel methods like comic-boarding, which is a co-design technique that uses comic strips as a framework to elicit ideas from populations who are not familiar with brainstorming or have lower literacy levels (33). As more AI healthcare tools are developed for pediatrics, it is critical to develop and apply design methods that allow patients/caregivers and medical team members are empowered to “drive or own any part of the design process itself” (31). There will likely be challenges to empowering users to drive a portion of the design process for novel intelligent tools that they likely have not experienced in specific clinical environments, like the ICU. Engaging pediatric populations is another unique challenge, but children and young people are interested in contributing to AI development and research in healthcare (34).

## User interface and interactions

Autonomy, transparency, explainability, and intelligibility have all been determined as key ethical principles by the World Health Organization (WHO), highlighting the importance of the user interface and interactions. Yet, while technical advances have improved LLM models, less attention has been paid to the design of the user interface and interaction experience—both of which are essential for supporting user understanding, trust, and accurate disclosure. Prior work in computer science and human-computer interaction has shown how the interface and interactions can be designed to optimize the abovementioned ethical principles. For example, Khurana et al. designed explainable chatbot interfaces that clarified both the chatbot's function and the reasons for any breakdowns, which increased perceptions of usefulness, transparency, and trust (35). Other research has shown that creating a chatbot with both a visual presence and voice interactions can lead to greater trust compared to chatbots with no visual component (36). This research highlights the need for deliberate interface and interaction design choices that go beyond backend model performance to center the user experience in ethically-aligned conversational AI systems. While there are a multitude of design considerations that researchers, designers, and developers may choose to contend within the creation of these tools, here we offer a few critical questions that will be imperative for designing these tools to uphold core ethical principles: How can conversational flows support user autonomy while ensuring the collection of complete and accurate medical information? What types of explanations—such as clarifying why specific questions are asked or how responses will be used—best promote intelligibility and trust? And how can interaction design help users accurately calibrate their trust in chatbot-collected histories, especially when the tool is embedded in clinical workflows?

## Workflow integration and output

One challenge to workflow integration is that there are no frameworks for clinical practice integration of chatbots. In addition, PICU workflows vary in the number and type of providers and patients; therefore, specific workflow designs may differ among institutions. We propose a few key areas of workflow integration to explore. For patients/caregivers, developers should understand their prior experience and preferred timing to interact with a chatbot. Would they be interested in sharing additional information at any point during the hospital stay or only upon admission? For the medical team, focus on the type, presentation (i.e., summarized or full conversation transcript), and use of information gathered. In the outpatient setting, chatbots used to obtain a preliminary history from patients before their arrival to the appointment were well-received by patients (37). Similarly, the chatbot could be first introduced to patients in the waiting room of the emergency department (E.D), much before they reach an ICU. Overall, there is a need for more research in clinical environments like the PICU to understand how to integrate a chatbot into the clinical workflow.

Workflow integration for providers will be a key aspect to determine the output of the chatbot. From our perspective, a useful chatbot output would be a summarized version of the patient-history that also presents potential diagnoses to allow clinicians to verify their diagnostic thoughts while mostly focusing on management. Ideally, this information would be translated directly into the medical record, possibly in the form of a note or under a separate tab. A generative AI chatbot that can propose most-likely diagnoses and allow the clinician to facilitate iterative reasoning would be the most useful to reduced cognitive burden. Training for clinicians will be required to emphasize that the intention of the tool is to augment, not replace their own hypothesis-based testing (38).

## Chatbot architecture, training, and data privacy

There are also a variety of chatbot architectures to use, including LLM vs. rule-based. Determining the best architecture will depend on your user population and goals for the chatbot. In our opinion, a LLM structure may be more beneficial because it can process an input from the patient and produce a more nuanced, contextualized response, rather than a rule-based architecture that includes pre-fixed responses. The model should be trained on a dataset that includes notes from the pediatric intensive care unit, diagnostic reasoning resources, and validated screening tools, such as for social determinants of health and baseline functional status.

It is important to note that open-source models, like ChatGPT cannot be directly used for patient care due to issues with data privacy and HIPPA concerns. There are hospital systems that are adapting open-sourced models to create HIPPA compliant chatbots (39). These models are considered HIPPA compliant because they encrypt protected health information (PHI) and do not train their models with any PHI. Finally, a key consideration for patients/caregivers participation regarding data privacy will be determining how to best communicate to a patient/caregiver at the bedside about where their information will be stored, how it will be used, and how will it be protected.

## Preventing misinformation

Given the importance of obtaining accurate information to prevent miscommunication, diagnostic bias, and maintain patient trust, implementing guardrails is crucial for safe and ethical use of chatbots. Guardrails are a set of filters, rules, or tools that set limits to machine learning models, including chatbots, in order to guide the model to function as expected (40). Guardrails can also help reduce “hallucinations” or “AI misinformation,” which are inaccurate, but seemingly plausible statements generated by a chatbot (41, 42). Misinformation that is communicated to the medical team could have massive implications for a patient's diagnosis and management.

There are templates for guardrails that can place ethical restraints on chatbots to help them interact appropriately and politely with users while avoiding harmful topics (43). Given the known inherent biases of many machine learning models in medicine, it is possible that the chatbot should avoid asking questions about race/ethnicity to prevent further bias for models trained on open-sourced datasets (44). Other guardrails to consider are the type of information that the chatbot should offer. For instance, a medical history-taking chatbot potentially should avoid providing diagnoses directly to patients/caregivers until reviewed by the medical team. A generative AI chatbot may require an authorization code to unlock AI-generated diagnostic suggestions to the medical team. Another option would be to have a separate diagnostic, HIPPA-compliant LLM where the patient's summarized history could be transferred to for diagnostic reasoning.

After a chatbot is deployed in clinical practice, patients/caregivers continue to play a key role in human-in-the-loop verification to reduce the production of misinformation. An example of this type of verification is demonstrated in a small pilot study by Ramjee et al. where a medical expert verified the answer produced from a LLM-based healthcare chatbot called, Cataractbot (45). The purpose was to reduce errors, hallucinations, and biases. A similar strategy for a bedside PICU would be to require approval or editing to the summarized history by the patient/caregiver prior to it being presented to the medical team.

## Chatbot evaluation

Currently, there are a lack of standardized methods to evaluate chatbots, particularly for healthcare chatbots (46). Abbasian et al. discuss model evaluation in terms of intrinsic and extrinsic factors. Intrinsic factors include surface-level language performance without adequate understanding or assessment of the semantics or clinical context. Extrinsic evaluation incorporates user satisfaction and the model's ability to properly function in a healthcare context (47). The authors propose a framework that includes creating an “environment” with 3 configuration components (confounding variables, prompt techniques, and evaluation methods). The purpose is to create a uniform evaluation approach, achieve the desired outputs, and establish guidelines for healthcare domains (47). To apply this framework to a PICU chatbot, the confounding variables would include the users (patients/families) and task-type definition (obtain history of present illness, past medical/family/social histories, baseline functional status, and social needs screening). There are a variety of LLM prompt techniques to choose from, but a history-taking chatbot in the PICU may benefit from incorporating chain-of-thought, role-based, instruction, and few-shot prompting techniques. Evaluation could include score-based metrics to measure completeness, empathy, safety, and hallucinations with comparison to current PICU notes or pre-generated history-scripts. Patients/caregivers and medical team members should be an integral part of the extrinsic evaluation of chatbots before and after their deployment, particularly for user satisfaction. The lack of guidelines with specific metrics that help reduce human bias is one of the major challenges with user evaluation of healthcare chatbots. Human evaluators should be trained on specific evaluation methods and tools to ensure consistent application. User satisfaction should focus on the interface and the chatbot's ability to interact ethically and empathetically. After implementation of a chatbot in a clinical setting, there should be continued evaluation of the model's extrinsic factors.

Understanding how the families and clinicians perceive the chatbot impacts the patient-clinician relationship can inform future designs and applications. It is also important to explore how chatbot-obtained information affects changes in clinical practice. Provider documentation could be evaluated before and after the implementation of chatbots to see if there is more information documented, i.e., via LLM coded topics or word counts. In addition, researchers could consider evaluating clinical outcomes before and after chatbot implementation, such as assessing the identification and management of social needs. Ultimately, standardized technical and qualitative methods for assessing healthcare chatbots are needed.

## Discussion

In the fast-paced PICU, chatbots could streamline history-taking by delivering more comprehensive information quickly, supporting clinical decisions, and shifting cognitive demands. Chatbots as primary history-takers and synthesizers of that information could augment the amount of type 1 thinking PICU providers rely on and excel at. Offloading the slower, more laborious Type 2 cognitive processes that require significant mental energy will allow clinicians more mental bandwidth to focus on complex management solutions. However, introducing these tools for history-taking and diagnostic management may have a significant impact on the development of these cognitive processes during medical training. Using generative AI tools, like chatbots, should be incorporated into medical school curricula so trainees learn how to use these tools to support the development of their diagnostic scripts and clinical reasoning while also learning how to critically evaluate their outputs to ensure safe and ethical use.

Designing effective PICU chatbots for history-taking and diagnostic support requires rigorous, user-center development and evaluation. As healthcare chatbots become more popular, there is a growing opportunity to involve patients/caregivers in their design and evaluation. Phillip Kellmeyer describes a range of participation levels for the development of medical AI systems from “no participation” to “beyond participation” in which participants exhibit self-organization and community-led research (48). Developing highly complex, yet equitable medical AI systems requires both technical expertise and an understanding of the needs and values of the target population. While community-led development would be the ultimate level of participation, it depends on building trust between researchers and community members, offering opportunities for all individuals to participate, and providing the community members with the necessary skills to engage in the research process (48). Currently, there are many challenges to this level of community participation in healthcare AI development, including the lack of methods for standardized evaluation of AI tools and unbiased participation recruitment, particularly for vulnerable populations like critically ill children. Overall, chatbots are becoming more ubiquitous in medical care and our everyday lives. Chatbot development for previously unexplored clinical settings like the PICU could provide a unique opportunity to apply and evaluate novel methods that allow users, such as patients/caregivers and clinicians, to be active participants in the critical conversations for the design of safe and effective AI tools for clinical practice.

## Data Availability

The original contributions presented in the study are included in the article/Supplementary Material, further inquiries can be directed to the corresponding author.
